# Comparative Effectiveness of Interventions for Global Cognition in Patients With Mild Cognitive Impairment: A Systematic Review and Network Meta-Analysis of Randomized Controlled Trials

**DOI:** 10.3389/fnagi.2021.653340

**Published:** 2021-06-18

**Authors:** Zijun Xu, Wen Sun, Dexing Zhang, Vincent Chi-Ho Chung, Regina Wing-Shan Sit, Samuel Yeung-Shan Wong

**Affiliations:** Jockey Club School of Public Health and Primary Care, The Chinese University of Hong Kong, Hong Kong, China

**Keywords:** cognition, mild cognitive impairment, network meta-analysis, randomized controlled trial, systematic review

## Abstract

**Background:** There is a lack of study comprehensively comparing the effects of all existing types of interventions on global cognition among patients with mild cognitive impairment (MCI).

**Aims:** To conduct a network meta-analysis to evaluate the effectiveness of different types of interventions in improving global cognition among MCI patients.

**Methods:** Randomized controlled trials (RCTs) assessing the effects of pharmacological or non-pharmacological interventions on the Mini-Mental State Examination (MMSE) in MCI patients were included. Two authors independently screened the studies and extracted the data. Random-effects network meta-analysis was used to synthesize the data. Results were summarized as mean difference (MD) and corresponding 95% CIs of MMSE in forest plots.

**Results:** Fifty RCTs with 5,944 MCI patients met the inclusion criteria and 49 were included in the network meta-analysis. Compared with the control group, cognition-based intervention (MD = 0.80, 95% CI 0.04–1.57), physical exercise (MD = 1.92, 95% CI 1.19–2.64), combined physical exercise and cognition-based intervention (MD = 1.86, 95% CI 0.60–3.12), and antioxidants (MD = 0.94, 95% CI 0.04–1.83) had positive effects on MMSE in participants with MCI. There was no significant difference between all other interventions included and the control group.

**Conclusions:** This study suggested that cognition-based intervention, physical exercise, combined physical exercise and cognition-based intervention, and antioxidants could be among the most effective interventions on global cognition in older adults with MCI. The availability, acceptability, and cost-effectiveness of interventions should also be taken into consideration when selecting interventions.

**Registration:** PROSPERO CRD42020171985.

## Introduction

Mild cognitive impairment (MCI) is an intermediate state between physiological aging and somatic and psychological disorders such as Alzheimer's disease and vascular dementia (Petersen et al., 2014). Along with the rapid aging of the population, cognitive decline has become increasingly prevalent among the older generation (Daffner, 2010). A recent study showed that the incidence of MCI was at least 22.5 per 1,000 person-years among older adults aged 75 years and above. MCI patients are also a high-risk group of developing dementia (Mitchell and Shiri-Feshki, 2009). As a result, effective strategies to delay the process of cognitive decline in older adults have become a geriatric care priority (Song et al., 2018).

Several kinds of interventions have been applied to this specific population to reduce their cognitive decline. Systematic reviews and meta-analyses have also been conducted to measure the effect of different types of intervention on global cognition among MCI patients, including physical exercise (Song et al., 2018), cognition-based intervention (Wang et al., 2014), repetitive transcranial magnetic stimulation (Chou et al., 2020), acupuncture (Lan et al., 2020), western medication (Matsunaga et al., 2019), Chinese herbal medicine (Dong et al., 2016), probiotic (Den et al., 2020), vitamin, and mineral supplementation (McCleery et al., 2018). However, most of the current meta-analyses only compared one intervention with a control group or only compared two different interventions. It is difficult to compare the results between these studies and identify the best intervention for practical use.

Network meta-analysis is a methodological approach that served as an extension of traditional meta-analysis (Lu and Ades, 2004; Salanti et al., 2008; Mills et al., 2013). It can compare multiple treatments for a disease or condition simultaneously whether the treatments have been compared head-to-head. This approach can also combine both direct and indirect evidence in a single analysis.

There have been some network meta-analyses conducted to measure the effect of different exercises or cognitive interventions on cognitive functions among MCI patients. Wang's study in 2019 comparing the effect of different exercises on global cognition in adults with MCI found that resistance exercise was the best intervention for MCI (Wang et al., 2019). Liang's network meta-analysis in 2019 examined the effect of cognitive stimulation, cognitive training, and cognitive rehabilitation. The results indicated that cognitive stimulation had the best effect on cognitive function for MCI patients and cognitive training also had a significant positive effect (Liang et al., 2019).

Studies were also conducted to compare different non-pharmacological therapies. Liang's network meta-analysis in 2018 compared the effect of physical exercise, music therapy, computerized cognitive training, and nutrition therapy on Mini-Mental State Examination (MMSE) among participants with Alzheimer's disease or MCI (Liang et al., 2018). The pooled results from 15 studies showed that physical exercise had significant positive effects on MMSE (Liang et al., 2018). Wang's study in 2020 found that cognitive stimulation was the best non-pharmacological therapy for MMSE in MCI patients, followed by physical exercise, multi-domain interventions, musical therapy, and cognitive training (Wang et al., 2020).

Currently, there was no network meta-analysis comprehensively comparing the effect of all existing types of intervention on global cognition in patients with MCI. Therefore, we conducted this network meta-analysis to evaluate the effectiveness of different types of interventions in randomized controlled trials (RCTs) in terms of improving global cognition, which was measured by MMSE, among MCI patients after combining the direct and indirect evidence. The results of this network-meta-analysis would be useful for identifying the most beneficial interventions and informing clinical practices for improving global cognition in MCI patients.

## Materials and Methods

This study was a systematic review and network meta-analysis of RCTs targeting global cognition among MCI patients. It has been registered at the PROSPERO International Prospective Register of Systematic Reviews (CRD42020171985). The study was conducted in accordance with the Preferred Reporting Items for Systematic Reviews and Meta-Analysis (PRISMA) guidelines for Network Meta-analysis (Hutton et al., 2015). Conclusion making was based on the GRADE approach (Brignardello-Petersen et al., 2020).

### Search Strategy and Study Selection

Relevant studies were identified on June 2020 using MEDLINE, EMBASE, Cochrane Library, CINAHL, PsycINFO, and PsycARTICLES searched from the included journals' inceptions. The search keywords were mild cognitive impairment, randomized controlled trial, and placebo. The full search strategies in each database are shown in Supplementary Table 1. We restricted all searches to human studies, titles, and abstracts. An additional search was performed by identifying relevant studies included in relevant systematic reviews and meta-analyses.

Potentially relevant studies were first identified from titles and abstracts by two reviewers (Z.X., W.S.) independently following the same standard. The reviewers contacted the authors if the full texts of relevant studies were not available from public databases. The two reviewers (Z.X., W.S.) independently reviewed the full text of potentially eligible studies. Any disagreement was resolved through discussion with a third reviewer (D.Z.).

### Inclusion and Exclusion Criteria

The inclusion criteria were following the PICOS criteria.

1. Population: Mild cognitive impairment (MCI) patients aged 50 years or above, assessed by clinical diagnosis, Peterson criteria, Clinical Dementia Rating (CDR), the Mini-Mental State Examination (MMSE), or Montreal Cognitive Assessment (MoCA);
2. Intervention: Any intervention targeting MCI patients, including but not limited to physical exercise, cognition-based intervention, different medications, and different dietary supplementations, as well as their combinations. The interventions were not limited to their dosage, duration, or intensity;
3. Comparison: Control group including placebo, no treatment, usual care, or any other intervention with the intervention type different from that of other groups;
4. Outcome: Mini-Mental State-Examination (MMSE) which measured the global cognition of the MCI patients;
5. Study design: RCTs with two or more arms.

We excluded studies (1) with no peer-reviewed full texts, (2) non-English, (3) having the same type of intervention for both intervention and control group, and (4) cross-over studies with no outcome data at the midpoint.

### Data Extraction and Risk-Of-Bias Assessment

Two reviewers (Z.X., W.S.) independently extracted the information on patients, interventions, and outcomes of all eligible studies from July 2020. Outcomes were extracted as the mean and SDs for MMSE score at baseline and post-intervention or change from baseline to post-intervention if any. Results from intention-to-treat analysis were extracted more preferentially than that from completer analyses if both were presented. The Cochrane Collaboration Recommendations assessment tool was used to evaluate the risk of bias for each study (Higgins et al., 2019). Two reviewers (Z.X., W.S.) independently assessed seven domains for risk of bias in the Cochrane Handbook for Systematic Reviews of Interventions: random sequence generation, allocation concealment, blinding of participants and personnel, blinding of outcomes assessment, incomplete outcome data, selective reporting, and other bias. Studies with all domains as low risk were considered as overall low risk; studies with at least one domain as high risk were considered as high risk; the remaining studies were considered as unclear risk (Gardner et al., 2016). Disagreements in data extraction and risk-of-bias assessment were resolved after discussion if necessary, together with a third reviewer (D.Z.).

### Statistical Analysis

The effect sizes for MMSE were the mean difference (MD) and SD from baseline to post-intervention. According to the Cochrane Handbook, SD of change was calculated from *p*-values, *t* statistics, SEs, or 95% CIs if it was not available from the study (Higgins et al., 2019). When SD was not able to be calculated, we used a correlation coefficient of 0.68 between baseline and post-intervention MMSE to estimate the missing data (Higgins et al., 2019). The correlation coefficient was calculated using the mean correlation of studies without missing data.

A network meta-analysis was conducted to combine the evidence of both direct and indirect comparative effectiveness. A network plot was generated to visualize the relative amount of the evidence on the included interventions targeting MCI (Chaimani et al., 2013). In the network plot, the nodes represented different intervention types, the size of which represented the number of participants in the intervention. Each line connecting two nodes represented direct comparisons between different interventions, with the thickness representing the number of studies involving in each comparison.

In neuropsychiatric and psychotherapeutic research, the control conditions vary widely, including waitlist control, treatment as usual, active comparator, no treatment control, pill placebo, and minimal treatment control, which may affect the effect sizes in conducting network-meta analysis (Gold et al., 2017; Doyle et al., 2019). The previous meta-analysis found that there was no difference in effect size between no-treatment controls, treatment as usual, and pill placebo (Mohr et al., 2014). Following another decision framework about control conditions in RCTs in psychiatry, we lumped no treatment, treatment as usual, and pill placebo as a non-active control group (Gold et al., 2017). Interventions in the same class with a similar mechanism were categorized as the same intervention type, such as medications in the class.

The network meta-analysis was performed using the network package in STATA (version 16.0) (Borenstein et al., 2010; Shim et al., 2017). The random-effects model was used, which is conservative and allowing for between-study heterogeneity of clinical and methodology (Borenstein et al., 2010). The MD and corresponding 95% CI were used to summarize the distributions of effect size in the forest plot.

Homogeneity of effect sizes was calculated using the *I*^2^ statistic, and publication bias was assessed using funnel plots for all direct comparisons (Chaimani et al., 2013). Node-splitting model was used to assess potential inconsistency between direct and indirect evidence from the same intervention comparison (Bucher et al., 1997; Dias et al., 2010). Subgroup analysis was conducted among different MCI types and different follow-up periods.

## Results

### Study Selection and Characteristics

**Figure 1:**
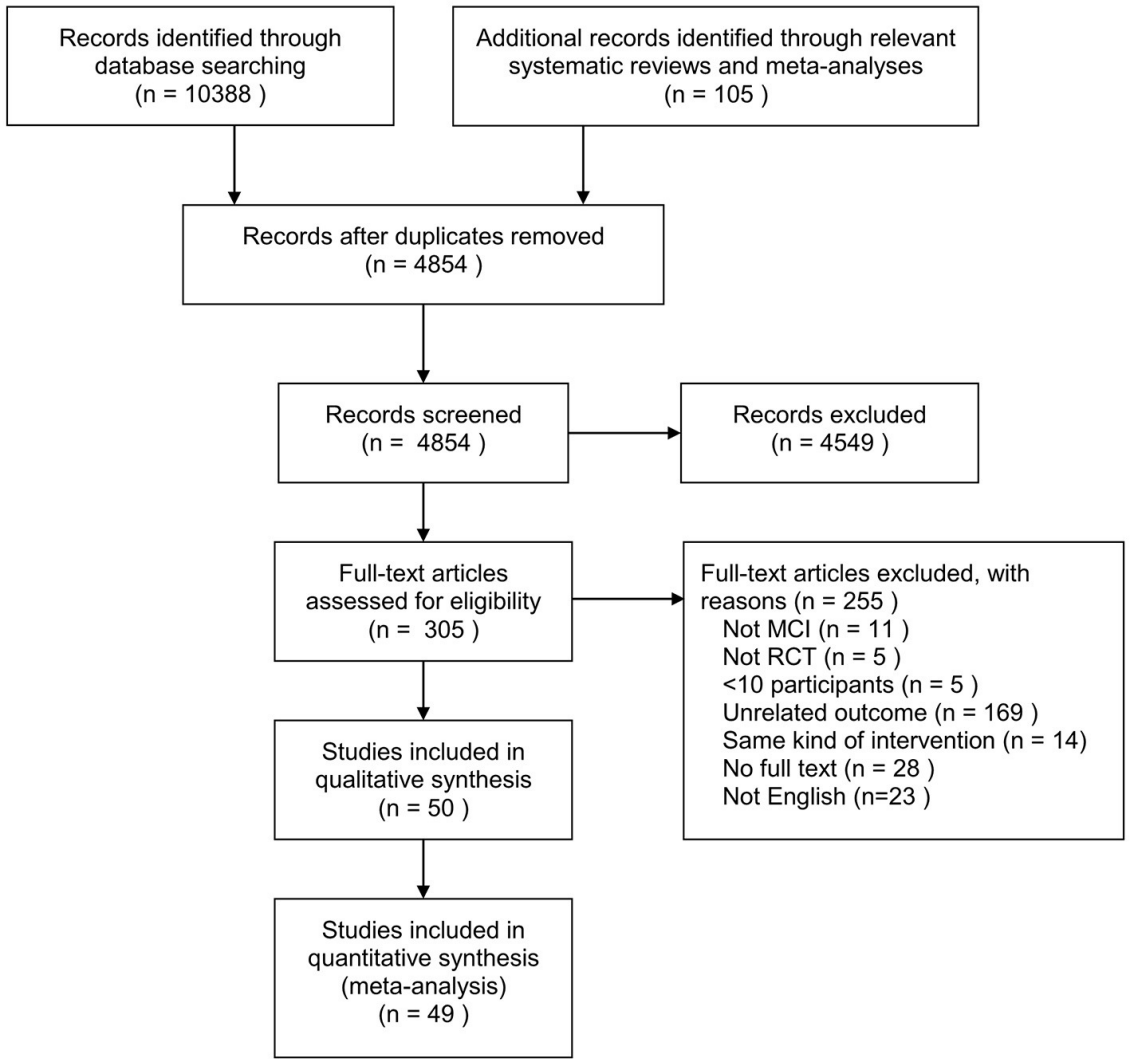
Flow chart of the study.

Figure 1 shows the flow diagram of the selection process. A total of 10,388 records were identified through database searching and 105 from relevant systematic reviews and meta-analyses. We reviewed 4,854 titles and abstracts for eligibility after removing duplicates. Fifty eligible RCTs were identified from 305 full-text reviews.

A total of 5,944 MCI patients were included in the 50 RCTs, with the sample size ranging from 21 to 757 participants included in the analysis. One study included naMCI (non-amnestic MCI) patients, 12 included only amnestic MCI (aMCI) patients, and 37 included MCI patients, among which two reported the results for aMCI patients. The mean age ranged from 61.7 to 85.8 years and the proportion of females ranged from 0 to 88%. The intervention and follow-up durations ranged from 1 to 48 months. Publication years ranged from 2005 until 2020. Supplementary Table 2 summarizes the characteristics of the included studies and Supplementary Table 3 presents the detailed intervention and effect size of all included studies.

Regarding the risk of bias, 6 (12%) studies had an overall low risk of bias, 27 (55%) had unclear risk, and 17 (33%) had a high risk of bias. The risk of bias was low for random sequence generation in 25 studies (50%), allocation concealment in 14 studies (28%), blinding of participants and personnel in 20 studies (40%), blinding of outcome assessment in 36 studies (72%), incomplete outcome data in 35 studies (70%), selective reporting in 50 studies (100%), and other bias in 50 studies (100%) (Supplementary Table 4 and Supplementary Figure 1).

### Network Meta-Analysis

**Figure 2:**
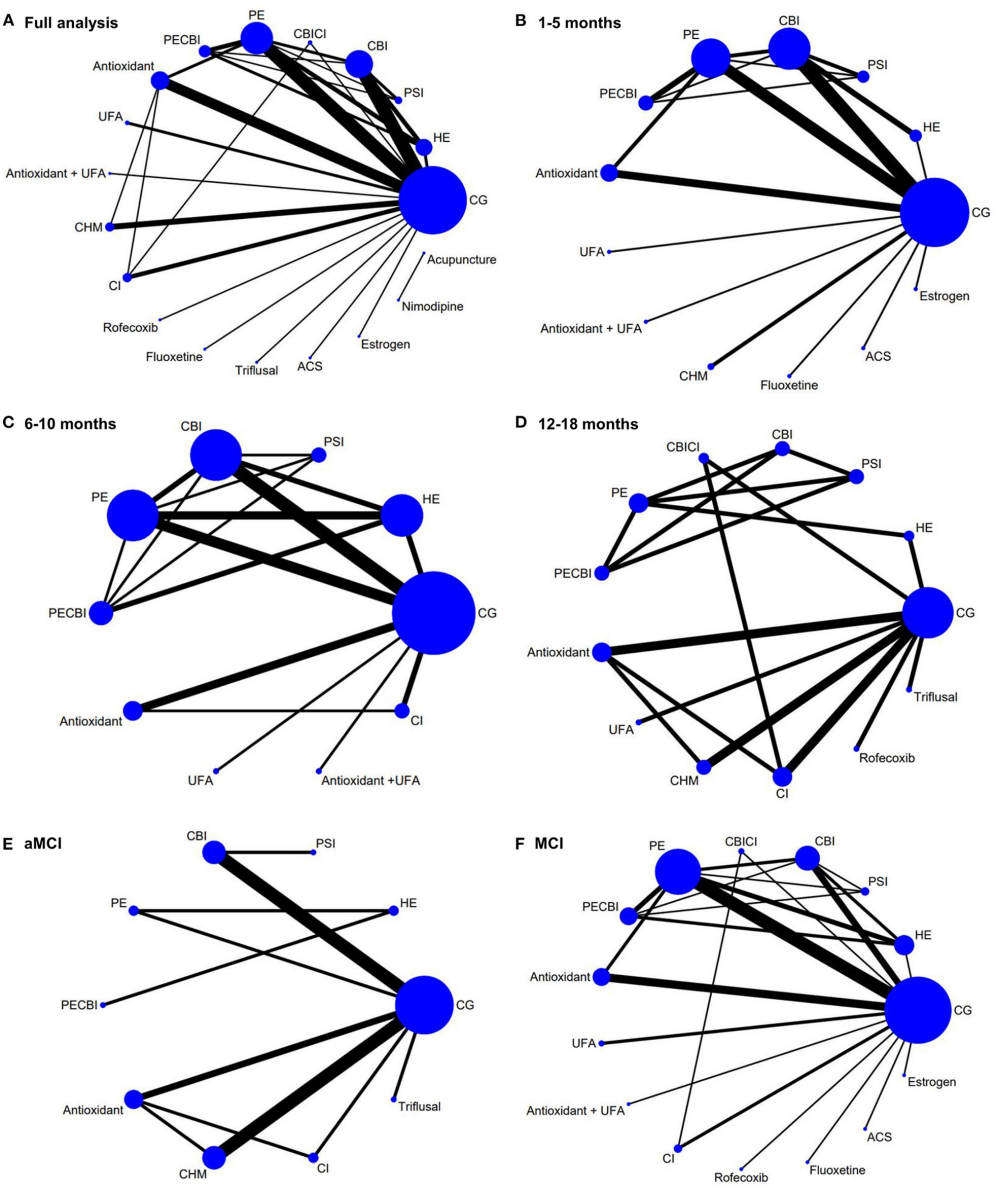
Network plot of included studies and subgroup analysis. **(A)** Network plot of the full analysis. **(B)** Network plot of the subgroup analysis in studies with a follow-up period of 1–5 months. **(C)** Network plot of the subgroup analysis in studies with a follow-up period of 6–10 months. **(D)** Network plot of the subgroup analysis in studies with a follow-up period of 12–18 months. **(E)** Network plot of the subgroup analysis in aMCI patients. **(F)** Network plot of the subgroup analysis in general MCI patients. The nodes represented different intervention types, the size of which represented the number of participants in the intervention. Each line connecting two nodes represented direct comparisons between different interventions, with the thickness representing the number of studies involving in each comparison. ACS, anserine/carnosine supplementation; CBI, cognition-based intervention; CBICI, cognition-based intervention and cholinesterase inhibitor; CG, control group; CHM, Chinese herbal medicine; CI, Cholinesterase inhibitor; HE, health education; PE, physical exercise; PECBI, physical exercise and cognition-based intervention; PSI, psychosocial intervention; UFA, unsaturated fatty acid.

**Figure 3:**
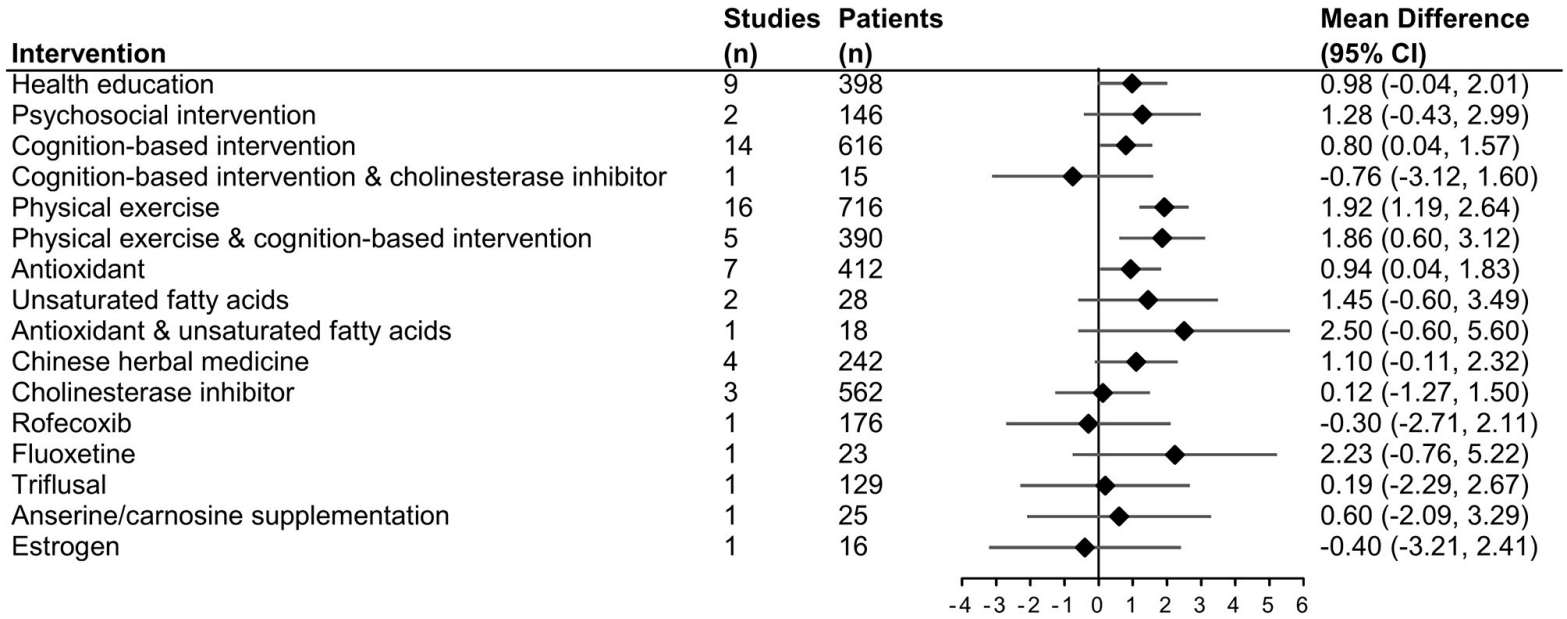
Effect size for change in MMSE compared with control group using forest plots.

Nineteen intervention types with 28 different comparisons were identified. One comparison between acupuncture and nimodipine did not connect with other comparisons. Therefore, the network meta-analysis included 49 RCTs and was conducted to compare the effectiveness of 17 intervention types with 27 different comparisons on MMSE. The network plot is shown in Figure 2A. The forest plot of pooled MDs for each intervention comparison is presented in Figure 3. Compared with the control group, cognition-based intervention (MD = 0.80, 95% CI 0.04–1.57), physical exercise (MD = 1.92, 95% CI 1.19–2.64), combined physical exercise and cognition-based intervention (MD = 1.86, 95% CI 0.60–3.12), and antioxidants (MD = 0.94, 95% CI 0.04–1.83) had positive effects on MMSE in participants with MCI. Compared with the control group, other intervention types all had non-significant on MMSE (Figure 3). The effect sizes of all direct and indirect comparisons are summarized in Supplementary Table 5.

The funnel plot in Supplementary Figure 2 shows no significant publication bias and some outliers show that heterogeneity existed. The overall network heterogeneity *I*^2^ was 93.2%. The node-splitting model assessing incoherence between direct and indirect comparisons showed there was no potential inconsistency between direct and indirect comparisons (*p* < 0.05) (Supplementary Table 6).

### Subgroup Analysis

**Figure 4:**
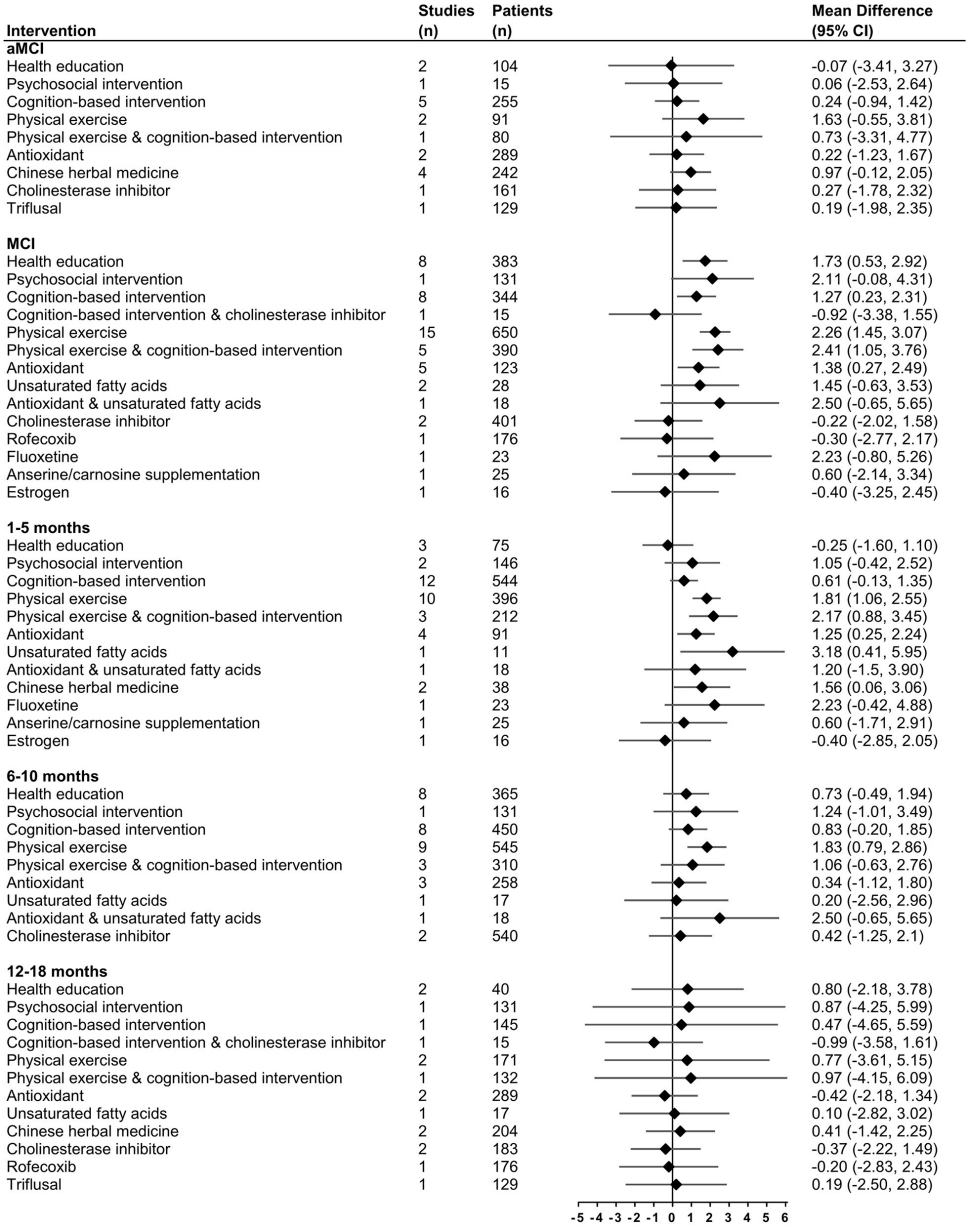
Effect size in subgroup analysis for change in MMSE compared with control group using forest plots.

One subgroup analysis was performed in studies with a follow-up period of 1–5, 6–10, and 12–18 months, respectively. The network plots are shown in Figures 2B–D. In studies with a follow-up period of 1–5 months, physical exercise (MD = 1.81, 95% CI 1.06–2.55), combined physical exercise and cognition-based intervention (MD = 2.17, 95% CI 0.88–3.45), antioxidants (MD = 1.25, 95% CI 0.25–2.24), unsaturated fatty acids (MD = 3.18, 95% CI 0.41–5.95), and Chinese herbal medicine (MD = 1.56, 95% CI 0.06–3.06) had a significant effect on MMSE (Figure 4). Physical exercise (MD = 1.83, 95% CI 0.79–2.86) had a significant effect in studies with a follow-up period of 6–10 months. Interventions were not significant in studies with a follow-up period of 12–18 months.

Another subgroup analysis was conducted among aMCI patients and the remaining studies included general MCI patients. The network plots are shown in Figures 2E,F. In aMCI patients, none of the interventions had a significant positive effect on MMSE. In the remaining MCI patients, health education (MD = 1.73, 95% CI 0.53–2.92), cognition-based intervention (MD = 1.27, 95% CI 0.23–2.31), physical exercise (MD = 2.26, 95% CI 1.45–3.07), combined physical exercise and cognition-based intervention (MD = 2.41, 95% CI 1.05–3.76), and antioxidants (MD = 1.38, 95% CI 0.27–2.49) all had significant effects on MMSE (Figure 4).

## Discussion

After performing a network meta-analysis to comparing the effects of all existing types of interventions on global cognition in MCI patients, the results found that cognition-based intervention, physical exercise, combined physical exercise and cognition-based intervention, and antioxidants had significant effects on global cognition in older adults with MCI when compared with the control group. We did not demonstrate any significant difference comparing other interventions with the control group. Subgroup analysis was conducted among different MCI types and follow-up periods of 1–5, 6–10, and 12–18 months. Some intervention types were reported by very limited studies; therefore, the results should be interpreted with caution.

Physical exercise and cognition-based intervention are the two most common interventions for MCI. Physical exercise combined with cognition-based intervention had an effect size of 1.86, which was comparable with physical exercise alone of 1.92 and beyond cognition-based intervention alone of 0.80. Compared with a recent network meta-analysis with four different interventions conducted among older adults with Alzheimer's disease or MCI, physical exercise ranked high and had significant positive effects on global cognition, and computerized cognitive training did not have a significant effect on cognition (Liang et al., 2018). The results in physical exercise were consistent with our study. A study found that among all kinds of physical exercises, resistance exercises, followed by resistance exercises and mind–body exercises, all had significant positive effects compared with the control (Wang et al., 2019). Cognition-based interventions in this study lumped all kinds of cognitive-based interventions. For the subtypes of cognitive intervention, a study found cognitive stimulation and cognitive training had positive effects on MMSE in MCI patients but cognitive rehabilitation had the lowest score (Liang et al., 2019). In terms of computerized cognitive training, there was still some debate on its effect, while most of the studies showed a positive impact on cognitive function (Hill et al., 2017; Liang et al., 2018; Hu et al., 2019; Zhang et al., 2019).

For medications and dietary supplementations, antioxidant supplements had significant effects on MMSE in MCI patients, which included antioxidant vitamins and plant extracts. In cohort studies, more antioxidant intake was associated with the improvement in cognitive domain scores and the reduced risk of cognitive decline (Maxwell et al., 2005; Nooyens et al., 2015). As people get older, more free radicals were produced to destroy tissue, including neurons. Antioxidant intake can help to neutralize these free radicals (Nooyens et al., 2015). Unsaturated fatty acids also had a moderate effect size, but only two RCTs were included and the 95% CI was large. A meta-analysis with seven RCTs about N−3 long-chain polyunsaturated fatty acids showed the potential benefit on cognition in older adults with MCI (Zhang et al., 2020). This network meta-analysis included one RCT measuring the effect of fluoxetine, which had a large effect size, but the result was not significant. Fluoxetine is a selective serotonin reuptake inhibitor that can promote neurogenesis in the hippocampus to prevent cognitive decline (Mowla et al., 2007). The current evidence on different nutrients, medications, and cognitive decline in a specific population of MCI is limited and more studies are needed.

The subgroup analysis showed different results for different MCI types and different follow-up durations. In the subgroup analysis of aMCI patients in nine RCTs, none of the interventions had a significant effect. However, in the remaining studies with general MCI patients, health education was regarded as an effective intervention for reducing cognitive decline. This indicated that interventions might be more difficult to work in aMCI patients. Patients with aMCI had lower hippocampus functional connectivity and higher functional connectivity, which were negatively correlated to episodic memory performance (Bai et al., 2009). Another trend we found is that with the increase of follow-up time, fewer interventions had positive and significant effects. Combined physical and cognition-based intervention, unsaturated fatty acids, and Chinese herbal medicine had short-term effects within a 5-month follow-up period, while physical exercise had a medium-to-long-term effect. It indicated that long-term adherence to interventions and reunion activities targeting cognitive decline might be needed for MCI patients. More studies that follow the participants for longer periods are needed as well.

The acceptability of the included interventions should also be taken into account. In a recent meta-analysis about the recruitment rate and adherence rate among MCI patients, the pooled intervention adherence rate for all types of interventions ranged from 82 to 96% (Xu et al., 2020). Transcranial magnetic stimulation, lifestyle modification/counseling, and dietary intervention had the top three intervention adherence rates of more than 90% in this study. Combined or single physical exercise and cognition-based study which were effective in this network meta-analysis had a moderate adherence rate of 84–88%. Medications had the lowest adherence rate of 76%. For interventions with moderate-to-low adherence, additional strategies should be applied such as counseling, additional educational sections, or simplifying the regimen (Doggrell, 2010; Marengoni et al., 2016). Future should also focus on the cost-effectiveness of the interventions and other outcomes in MCI patients such as depression, anxiety, quality of life, and physical activity.

To our knowledge, this study conducted a highly detailed literature search and evaluated all existing types of interventions conducted among MCI patients in a network meta-analysis to assess their MMSE. When interpreting the results, some limitations in this study should be taken into account. First, we did not include the scales measuring global cognition other than MMSE, such as Montreal Cognitive Assessment or Alzheimer's Disease Assessment Scale-Cognitive section. It was because standard mean difference (SMD) should be used to pool the effects of all scales of global cognition, which largely rely on the SDs of the score change at the end of the intervention. However, almost half of the SDs need to be estimated thus might affect the accuracy of SMD, so only MMSE was used as the outcome in this study. Second, there was high heterogeneity in the network meta-analysis due to methodological heterogeneity across the studies, such as different intervention regimens and different follow-up periods. The heterogeneity may impact the accuracy of the network meta-analysis, and the transitivity and consistency assumptions. Third, some intervention types, especially some medications and dietary supplements such as rofecoxib, fluoxetine, triflusal, anserine/carnosine, estrogen, and unsaturated fatty acids, were reported by very limited studies; therefore, the results should be treated with caution. Finally, although we conducted a comprehensive search on the topic, limited studies had direct comparisons between two or more active interventions. It indicates that more direct comparisons should be assessed in future RCTs.

## Conclusion

In conclusion, this network meta-analysis suggested that cognition-based intervention, physical exercise, combined physical exercise and cognition-based intervention, and antioxidants could be among the most effective interventions on global cognition in older adults with MCI. The results could inform future clinical and healthcare practice worldwide for preventing cognitive decline in MCI patients. Factors, such as the availability, acceptability, and cost-effectiveness of interventions, should be in consideration for treatment selection.

## Data Availability Statement

The original contributions presented in the study are included in the article/Supplementary Material, further inquiries can be directed to the corresponding author/s.

## Author Contributions

ZX, WS, DZ, and SW: conceptualization. ZW and WS: methodology. ZX: formal analysis, investigation, and writing—original draft preparation. DZ, VC, RS, and SW: writing—review and editing. SW: supervision. All authors contributed to the article and approved the submitted version.

## Conflict of Interest

The authors declare that the research was conducted in the absence of any commercial or financial relationships that could be construed as a potential conflict of interest.
