# Supplementary material for: Comparative Effectiveness of Interventions for Global Cognition in Patients With Mild Cognitive Impairment: A Systematic Review and Network Meta-Analysis of Randomized Controlled Trials
Source: Front Aging Neurosci. 2021 Jun 18;13:653340. doi: 10.3389/fnagi.2021.653340 (PMC8249717; doi:10.3389/fnagi.2021.653340)
Supplement: Supplementary file 1 [file Table_1.DOCX]

Supplementary Material

**Supplementary Table 1.** Searching strategy (limit to title/abstract and human research)

| Database | Searching strategy | Result |
| --- | --- | --- |
| MEDLINE | ((“mild cognitive impairment”)[Title/Abstract] AND (Trial*[Title/Abstract] OR random*[Title/Abstract] OR controlled clinical trial[Title/Abstract] OR placebo)[Title/Abstract]) | 1948 |
| Cochrane Library | ((“mild cognitive impairment”) AND (Trial* OR random* OR controlled clinical trial OR placebo)):ti,ab | 1896 |
| CINAHL | S1 TI (“mild cognitive impairment”) AND (Trial* OR random* OR controlled clinical trial OR placebo)  S2 AB (“mild cognitive impairment”) AND (Trial* OR random* OR controlled clinical trial OR placebo)  S3 S1 OR S2 | 1192 |
| EMBASE | (mild cognitive impairment and (Trial* or random* or controlled clinical trial or placebo)).ab,ti. | 4061 |
| PsycINFO | (mild cognitive impairment and (Trial* or random* or controlled clinical trial or placebo)).ab,ti. | 1280 |
| PsycARTICLES | (mild cognitive impairment and (Trial* or random* or controlled clinical trial or placebo)).ab,ti. | 11 |

**Supplementary Table 2.** Characteristics of included studies

| Study | Country | Participants | | | | Intervention | | Follow-up duration (months) |
| --- | --- | --- | --- | --- | --- | --- | --- | --- |
|  |  | MCI type | N | Age (mean) | Female (%) | Type of intervention | Duration (months) |  |
| Petersen (2005)(1) | USA, Canada | aMCI | 539 | 72.9 | 46 | Antioxidant vs CI vs CG | 36 | 6/12/18/24/30/36 |
| Thal (2005)(2) | USA | MCI | 371 | 74.9 | 33 | Rofecoxib vs CG | 48 | 12/24/36/48 |
| Mowla (2007)(3) | Iran | MCI | 44 | 63.4 | 57 | Fluoxetine vs CG | 2 | 2 |
| Rozzini (2007)(4) | Italy | MCI | 59 | NA | NA | CBICI vs CI vs CG | 12 | 12 |
| Gómez-Isla (2008)(5) | Spain, Portugal | aMCI | 257 | 73.2 | 66 | Triflusal vs CG | 18 | 18 |
| Doody (2009)(6) | USA | MCI | 757 | 70.0 | 46 | CI vs CG | 8 | 8 |
| Kwok (2011)(7) | China (Hong Kong) | MCI | 31 | 85.8 | 80 | CBI vs CG | 2 | 2 |
| Sherwin (2011)(8) | Canada | MCI | 28 | 75.9 | 0 | Estrogen vs CG | 3 | 3 |
| Rondanelli (2012)(9) | Italy | MCI | 25 | 85.7 | 80 | UFA vs CG | 3 | 3 |
| Suzuki (2012)(10) | Japan | (a)MCI | 47 | 76.1 | 46 | PE vs HE | 12 | 6/12 |
| Varela (2012)(11) | Spain | MCI | 63 | 78.2 | 56 | PE vs CBI | 3 | 3/6 |
| Lee (2013)(12) | Malaysia | MCI | 35 | 64.9 | 77 | UFA vs CG | 12 | 6/12 |
| Rojas (2013)(13) | Argentina | MCI | 30 | 74.5 | 43 | CBI vs CG | 6 | 12 |
| Suzuki (2013)(14) | Japan | MCI | 92 | 75.4 | 51 | PE vs HE | 6 | 6 |
| Yakoot (2013)(15) | Egypt | MCI | 60 | 66.2 | 45 | Antioxidant vs CG | 1 | 1 |
| Zhang (2013)(16) | China | MCI | 233 | 71.3 | 53 | Nimodipine vs Acupuncture | 2 | 2 |
| Johari (2014)(17) | Malaysia | MCI | 35 | 64.7 | 54 | HE vs CG | 12 | 6/12 |
| Wei (2014)(18) | China | MCI | 60 | 66.0 | 33 | PE vs CG | 6 | 3/6 |
| Zhang (2014)(19) | China | aMCI | 39 | 61.7 | 54 | CHM vs CG | 3 | 3 |
| Ciarmiello (2015)(20) | Italy | aMCI | 30 | 71.6 | 59 | CBI vs PSI | 4 | 4 |
| Zhang (2015)(21) | China | aMCI | 44 | 63.7 | 48 | CHM vs CG | 3 | 3 |
| Lam (2015)(22) | China (Hong Kong) | MCI | 555 | 75.4 | 78 | PE vs CBI vs PECBI vs PSI | 12 | 4/8/12 |
| Barban (2016)(23) | Italy, Greece, Norway, Spain | MCI | 106 | 73.6 | 47 | CBI vs CG | 3 | 3 |
| Barekatain (2016)(24) | Iran | naMCI | 51 | 65.3 | 88 | CBI vs HE vs CG | 4 | 2/6 |
| Giuli (2016)(25) | Italy | MCI | 97 | 76.3 | 62 | CBI vs CG | 2.5 | 2.5 |
| Hagovska (2016)(26) | Slovak Republic | MCI | 78 | 67.0 | 49 | PECBI vs PE | 2.5 | 2.5 |
| Jeong (2016)(27) | Korea | aMCI | 224 | 70.3 | 63 | CBI vs CG | 3 | 3/9 |
| Zhang (2016)(28) | China | aMCI | 60 | 64.7 | 53 | CHM vs CG | 24 | 12/24 |
| Doi (2017)(29) | Japan | MCI | 201 | 75.9 | 52 | PE vs HE | 10 | 10 |
| Hagovska (2017)(30) | Slovak Republic | MCI | 80 | 67.1 | 49 | PECBI vs PE | 2.5 | 2.5 |
| Kohanpour-1 (2017)(31) | Iran | MCI | 40 | 68.1 | NA | PE vs Antioxidant vs CG | 3 | 3 |
| Kohanpour-2 (2017)(32) | Iran | MCI | 40 | 67.9 | NA | PE vs Antioxidant vs CG | 3 | 3 |
| Lazarou (2017)(33) | Greece | aMCI | 129 | 66.8 | 79 | PE vs CG | 10 | 10 |
| Poptsi (2018)(34) | Greece | aMCI | 71 | 68.5 | 69 | CBI vs CG | 6 | 6 |
| Shimada (2018)(35) | Japan | (a)MCI | 266 | 71.6 | 50 | PECBI vs HE | 10 | 10 |
| Qi (2019)(36) | China | MCI | 32 | 69.9 | 72 | PE vs CG | 3 | 3 |
| Bademli (2019)(37) | Turkey | MCI | 60 | 71.5 | 58 | PE vs CG | 5 | 5 |
| Bae (2019)(38) | Japan | MCI | 83 | 76.0 | 48 | PECBI vs HE | 6 | 6 |
| de Oliveira Silva (2019)(39) | Brazil | MCI | 28 | 75.0 | 58 | PE vs CG | 3 | 3 |
| Klainin-Yobas (2019)(40) | Singapore | MCI | 55 | 71.3 | 75 | CBI vs HE | 9 | 3/9 |
| Langoni (2019)(41) | Brazil | MCI | 52 | 72.3 | 77 | PE vs CG | 6 | 6 |
| Masuoka (2019)(42) | Japan | MCI | 50 | 73.3 | 52 | ACS vs CG | 3 | 3 |
| Park-1 (2019)(43) | South Korea | MCI | 45 | 71.6 | 69 | PE vs CG | 6 | 6 |
| Park-2 (2019)(44) | South Korea | aMCI | 50 | 70.2 | 56 | CBI vs CG | 3 | 3/6 |
| Park-3 (2019)(45) | South Korea | MCI | 82 | 62.2 | 67 | Antioxidant vs CG | 6 | 4/6 |
| Tadokoro (2019)(46) | Japan | MCI | 59 | 75.7 | 51 | Antioxidant vs CG | 6 | 6 |
| Tian (2019)(47) | China | aMCI | 348 | 63.7 | 51 | CHM vs Antioxidant vs CG | 12 | 12 |
| Park (2020)(48) | South Korea | aMCI | 21 | 70.6 | 67 | CBI vs CG | 3 | 3 |
| Stavrinou (2020)(49) | Cyprus | MCI | 36 | 78.8 | 61 | Antioxidant+UFA vs CG | 6 | 3/6 |
| Thapa (2020)(50) | South Korea | MCI | 66 | 72.5 | 79 | CBI vs HE | 2 | 2 |

ACS: anserine/carnosine supplementation; aMCI: amnestic mild cognitive impairment; (a)MCI: MCI patients included and results for aMCI patients reported; CBI: cognition-based intervention; CBICI: cognition-based intervention and cholinesterase inhibitor; CG: control group; CHM: Chinese herbal medicine; CI: Cholinesterase inhibitor; HE: health education; MCI: mild cognitive impairment; NA: not available; naMCI: non-amnestic mild cognitive impairment; PE: physical exercise; PECBI: physical exercise and cognition-based intervention; PSI: psychosocial intervention; UFA: unsaturated fatty acid.

**Supplementary Table 3.** Description of interventions and effect size of MSSE change for all included studies

| Study | Description of interventions | Mean MMSE change |
| --- | --- | --- |
| Petersen (2005)(1) | Group 1: Vitamin E (1000 IU/d, first 6w; 2000 IU/d, after 6w), placebo donepezil, multivitamin Group 2: Donepezil (5 mg/d, first 6w; 10 mg/d, after 6w), placebo vitamin E, multivitamin Group 3: Placebo vitamin E, placebo donepezil, multivitamin | Group 1: -2.20±3.64  Group 2: -2.31±3.72 Group 3: -2.75±4.04 |
| Thal (2005)(2) | Group 1: Rofecoxib (25 mg/d) Group 2: Placebo | Group 1: -0.9±1.57 Group 2: -0.6±1.57 |
| Mowla (2007)(3) | Group 1: Fluoxetine (10 mg/d first 1-2w; 20 mg/d, 1-2w later) Group 2: Placebo | Group 1: 2.83±4.13 Group 2: 0.6±1.37 |
| Rozzini (2007)(4) | Group 1: NeuroPsychological Training + cholinesterase inhibitors  Group 2: Cholinesterase inhibitors  Group 3: No treatment | Group 1: -0.8±1.49 Group 2: -0.3±1.98 Group 3: 0.3±1.53 |
| Gómez-Isla (2008)(5) | Group 1: Triflusal (900 mg/d)  Group 2: Placebo | Group 1: -0.538±2.68 Group 2: -0.727±2.72 |
| Doody (2009)(6) | Group 1: Donepezil (5-10 mg/d)  Group 2: Placebo | Group 1: 0.1±0.2 Group 2: 0.0±0.2 |
| Kwok (2011)(7) | Group 1: Calligraphy (30 min/d, 5 d/w) Group 2: No intervention | Group 1: 2.36±2.59 Group 2: −0.41±2.50 |
| Sherwin (2011)(8) | Group 1: Micronized E2 Group 2: Placebo | Group 1: -0.1±2.06 Group 2: 0.3±1.88 |
| Rondanelli (2012)(9) | Group 1: Oily emulsion of DHA-phospholipids containing melatonin and tryptophan (2 capsules/d) Group 2: Placebo | Group 1: 1.01±1.89 Group 2: -2.17±3.07 |
| Suzuki (2012)(10) | Group 1: Multicomponent exercise (90 min/d, 2 d/w, 80 times) Group 2: Education control | Group 1:−0.47±3.20 Group 2: −0.44±3.32 |
| Varela (2012)(11) | Group 1: Aerobic exercise (40% HRR, 30 min/d, 3 d/w) Group 2: Aerobic exercise (60% HRR, 30 min/d, 3 d/w) Group 3: Recreational activities (30 min/d, 3 d/w) | Group 1: 1.09±1.97 Group 2: 0.42±0.29 Group 3: -2.27±2.27 |
| Lee (2013)(12) | Group 1: Concentrated DHA fish oil: DHA (3*430 mg/d), EPA (3*150 mg/d) Group 2: Placebo | Group 1: 0.2±1.98 Group 2: 0.1±1.97 |
| Rojas (2013)(13) | Group 1: Cognitive intervention program  Group 2: No intervention | Group 1: 0±1.76 Group 2: -1.77±1.89 |
| Suzuki (2013)(14) | Group 1: Multicomponent exercise (90 min/d, 2 d/w) Group 2: Education control group | Group 1: 0.2±2.62 Group 2: -0.3±2.57 |
| Yakoot (2013)(15) | Group 1: Memo (1 capsule/d) Group 2: Placebo | Group 1: 2.067±1.143 Group 2: 0.133±1.167 |
| Zhang (2013)(16) | Group 1: Nimodipine Group 2: Scalp electroacupuncture Group 3: Syndrome differentiation | Group 1: 2.3±1.03 Group 2: 3.3±1.03 Group 3: 3.6±0.95 |
| Johari (2014)(17) | Group 1: Nutrition and lifestyle education (1 d/m) Group 2: Placebo | Group 1: 1.6±2.5 Group 2: 0.8±1.8 |
| Wei (2014)(18) | Group 1: Handball training (30 min/d, 5 d/w) Group 2: No intervention | Group 1: 1.2±1.25 Group 2: -0.33±1.09 |
| Zhang (2014)(19) | Group 1: Congrongyizhi Capsule Group 2: Placebo Group 3: No treatment | Group 1: 1.5±1.14 Group 2: 0.5±1.55 Group 3: -0.62±1.21 |
| Ciarmiello (2015)(20) | Group 1: Cognitive stimulation training (45 min/d, 2 d/w) Group 2: Informal meeting with a psychologist (45 min/d, 2 d/w) | Group 1: 0.39±1.37 Group 2: 0.21±1.48 |
| Zhang (2015)(21) | Group 1: Bushen capsule (3*300 mg/d) Group 2: Placebo | Group 1: 1.09±1.35 Group 2: -0.4±1.4 |
| Lam (2015)(22) | Group 1: Physical exercise (1 h/d, 3 d/w) Group 2: Cognitive activity (1 h/d, 3 d/w) Group 3: Integrated cognitive and physical exercise (1 h/d, 3 d/w) Group 4: Social activity (1 h/d, 3 d/w) | Group 1: -0.2±1.88 Group 2: -0.5±2.17 Group 3: 0±2.15 Group 4: -0.1±1.96 |
| Barban (2016)(23) | Group 1: Process-based cognitive training (pb-CT) combined with reminiscence therapy (RT) Group 2: Rest | Group 1: 0.6±1.61 Group 2: -0.4±1.27 |
| Barekatain (2016)(24) | Group 1: Cognitive rehabilitation (2 h/w) Group 2: Lifestyle modification  Group 3: Control | Group 1: 0.53±1.09 Group 2: -0.7±1.12 Group 3: 0.77±1.49 |
| Giuli (2016)(25) | Group 1: Comprehensive cognitive training Group 2: General psychoeducational approach | Group 1: -0.23±1.84 Group 2: -0.42±2.35 |
| Hagovska (2016)(26) | Group 1: Cognitive training (CogniPlus program combined with balance training) Group 2: Balance training | Group 1: 1±1.94 Group 2: 0.08±1.17 |
| Jeong (2016)(27) | Group 1: Group-based cognitive intervention (2 d/w) Group 2: Home-based cognitive intervention (5 d/w) Group 3: No intervention | Group 1: 0.3±1.8 Group 2: 0.7±2.0 Group 3: 0.3±1.8 |
| Zhang (2016)(28) | Group 1: Xingnao granules 3 times a day, 4 capsules a time Group 2: Placebo 3 times a day, 4 capsules a time | Group 1: 0.68±1.5 Group 2: -1.32±2.29 |
| Doi (2017)(29) | Group 1: Dance (40 min/w) Group 2: Playing musical instruments (40 min/w) Group 3: Health education control group | Group 1: 0.29±2.6 Group 2: 0.46±2.1 Group 3: -0.36±2.3 |
| Hagovska (2017)(30) | Group 1: Physical training (30 min/d) + CogniPlus  Group 2: Physical training (30 min/d) | Group 1: 0.99±1.94 Group 2: -0.01±1.16 |
| Kohanpour-1 (2017)(31) | Group 1: Aerobic exercise, Glycyrrhiza glabra extract  Group 2: Aerobic exercise Group 3: Glycyrrhiza glabra extract  Group 4: Placebo | Group 1: 3±1.17 Group 2: 1.7±1.24 Group 3: 1.6±1 Group 4: -0.1±0.7 |
| Kohanpour-2 (2017)(32) | Group 1: Aerobic exercise, lavender extract Group 2: Aerobic exercise Group 3: Lavender extract Group 4: Placebo | Group 1: 2.4±0.99 Group 2: 1.7±1.24 Group 3: 1.5±1.29 Group 4: -0.1±0.69 |
| Lazarou (2017)(33) | Group 1: International Ballroom Dancing class twice a week  Group 2: No intervention | Group 1: 0.4±1.72 Group 2: -1.23±2.4 |
| Poptsi (2018)(34) | Group 1: Computer-based program of language tasks group Group 2: Oral group  Group 3: Passive control group  Group 4: Active control group  Group 5: Paper and pencil group | Group 1: 0.85±1.21 Group 2: -0.2±2.01 Group 3: 0.85±2.33 Group 4: 0.2±1.57 Group 5: 0.44±1.06 |
| Shimada (2018)(35) | Group 1: Cognitive and physical activity (90 min/w) Group 2: Health education program (90 min/w) | Group 1: 0.0±2.39 Group 2: -0.8±2.32 |
| Qi (2019)(36) | Group 1: Exercise (aerobic dance intervention) +usual care Group 2: Usual care control | Group 1: 0.9±1.2 Group 2: 0.2±2.1 |
| Bademli (2019)(37) | Group 1: Physical exercises (80min/d, 3-7d/w) Group 2: Daily routine activities | Group 1: 3.272±1.63 Group 2: -1.18±0.89 |
| Bae (2019)(38) | Group 1: Multicomponent intervention group (physical, cognitive, or social activity, 90 min/d, 2 d/w) Group 2: Health education | Group 1: −0.51±2.76 Group 2: 0.35±2.63 |
| de Oliveira Silva (2019)(39) | Group 1: Multimodal physical exercises (aerobic, strength, balance and ﬂexibility) (60 min/d, 2 d/w) Group 2: Control | Group 1: -0.5±0.87 Group 2: -0.75±1.16 |
| Klainin-Yobas (2019)(40) | Group 1: Mindful awareness program (60 min/d) Group 2: Health education | Group 1: -0.36±2.38 Group 2: 0.67±2.88 |
| Langoni (2019)(41) | Group 1: Strength and aerobic exercise (60 min/d, 2 d/w) Group 2: No intervention | Group 1: 3.1±2.4 Group 2: -3.3±1.9 |
| Masuoka (2019)(42) | Group 1: Anserine (750 mg/d), Carnosine (250 mg/d) Group 2: Placebo | Group 1: -0.4±1.9 Group 2: -1±2.5 |
| Park-1 (2019)(43) | Group 1: Dual-Task Exercise Program Group 2: No intervention | Group 1: 0.8±2.71 Group 2: -0.2±2.44 |
| Park-2 (2019)(44) | Group 1: Home based cognitive intervention  Group 2: No intervention | Group 1: 0.1±2.4 Group 2: -1.2±1.9 |
| Park-3 (2019)(45) | Group 1: Ginseng (53 mg/g ginsenoside) Group 2: Placebo | Group 1: 0.61±1.38 Group 2: 0.54±1.68 |
| Tadokoro (2019)(46) | Group 1: Antioxidative supplement Twendee X  Group 2: Placebo | Group 1: 0.66±2.60 Group 2: -0.85±2.48 |
| Tian (2019)(47) | Group 1: Qinggongshoutao Group 2: EGb761, Ginkgo biloba extract Group 3: Placebo | Group 1: -0.92±1.85 Group 2: -0.86±1.84 Group 3: 0.25±1.84 |
| Park (2020)(48) | Group 1: Virtual Reality based training group  Group 2: No intervention | Group 1: 0.5±2.08 Group 2: 0.64±1.5 |
| Stavrinou (2020)(49) | Group 1: Omega-3, omega-6 fatty acids, and antioxidant Vitamins  Group 2: Placebo | Group 1: 1.8±2.64 Group 2: -0.7±3.37 |
| Thapa (2020)(50) | Group 1: Virtual Reality based training group (100min/w, 3d/w) Group 2: Educational program on general health care (1d/w) | Group 1: 0.9±1.53 Group 2: 0.1±2.46 |

**Supplementary Table 4.** Risk of bias of included trials evaluated using the Cochrane Risk of Bias tool

| Study | Random sequence generation (selection bias) | Allocation concealment (selection bias) | Blinding of participants and personnel (performance bias) | Blinding of outcome assessment (detection bias) | Incomplete outcome data (attrition bias) | Selective reporting (reporting bias) | Other bias | Overall bias |
| --- | --- | --- | --- | --- | --- | --- | --- | --- |
| Petersen (2005)(1) | unclear | unclear | low | low | low | low | low | unclear |
| Thal (2005)(2) | low | low | low | low | low | low | low | low |
| Mowla (2007)(3) | unclear | unclear | low | low | unclear | low | low | unclear |
| Rozzini (2007)(4) | unclear | unclear | unclear | low | low | low | low | unclear |
| Gómez-Isla (2008)(5) | unclear | unclear | low | low | low | low | low | unclear |
| Doody (2009)(6) | low | low | low | low | low | low | low | low |
| Kwok (2011)(7) | unclear | unclear | high | low | low | low | low | high |
| Sherwin (2011)(8) | unclear | unclear | low | low | unclear | low | low | unclear |
| Rondanelli (2012)(9) | low | low | low | low | low | low | low | low |
| Suzuki (2012)(10) | unclear | unclear | unclear | low | low | low | low | unclear |
| Varela (2012)(11) | unclear | unclear | unclear | low | low | low | low | unclear |
| Lee (2013)(12) | low | low | low | low | low | low | low | low |
| Rojas (2013)(13) | unclear | unclear | high | high | high | low | low | high |
| Suzuki (2013)(14) | low | low | unclear | low | low | low | low | unclear |
| Yakoot (2013)(15) | low | low | low | low | low | low | low | low |
| Zhang (2013)(16) | unclear | unclear | unclear | unclear | unclear | low | low | unclear |
| Johari (2014)(17) | unclear | unclear | high | unclear | low | low | low | high |
| Wei (2014)(18) | unclear | unclear | unclear | unclear | unclear | low | low | unclear |
| Zhang (2014)(19) | unclear | unclear | low | low | low | low | low | unclear |
| Ciarmiello (2015)(20) | unclear | unclear | unclear | unclear | low | low | low | unclear |
| Zhang (2015)(21) | unclear | unclear | low | low | low | low | low | unclear |
| Lam (2015)(22) | low | unclear | high | low | low | low | low | high |
| Barban (2016)(23) | low | low | unclear | low | unclear | low | low | unclear |
| Barekatain (2016)(24) | low | unclear | low | low | low | low | low | unclear |
| Giuli (2016)(25) | low | unclear | unclear | unclear | unclear | low | low | unclear |
| Hagovska (2016)(26) | low | low | low | unclear | unclear | low | low | unclear |
| Jeong (2016)(27) | low | low | high | low | low | low | low | high |
| Zhang (2016)(28) | unclear | unclear | low | low | low | low | low | unclear |
| Doi (2017)(29) | unclear | unclear | high | low | low | low | low | high |
| Hagovska (2017)(30) | low | low | unclear | low | low | low | low | unclear |
| Kohanpour-1 (2017)(31) | unclear | unclear | unclear | unclear | low | low | low | unclear |
| Kohanpour-2 (2017)(32) | unclear | unclear | unclear | unclear | low | low | low | unclear |
| Lazarou (2017)(33) | low | low | high | low | high | low | low | high |
| Poptsi (2018)(34) | low | low | high | low | low | low | low | high |
| Shimada (2018)(35) | low | unclear | high | low | low | low | low | high |
| Qi (2019)(36) | unclear | unclear | low | low | low | low | low | unclear |
| Bademli (2019)(37) | low | unclear | high | high | low | low | low | high |
| Bae (2019)(38) | low | unclear | high | low | low | low | low | high |
| de Oliveira Silva (2019)(39) | unclear | unclear | high | low | high | low | low | high |
| Klainin-Yobas (2019)(40) | low | unclear | high | high | low | low | low | high |
| Langoni (2019)(41) | low | unclear | high | low | unclear | low | low | high |
| Masuoka (2019)(42) | unclear | unclear | low | low | low | low | low | unclear |
| Park-1 (2019)(43) | unclear | unclear | high | low | low | low | low | high |
| Park-2 (2019)(44) | low | low | high | high | high | low | low | high |
| Park-3 (2019)(45) | unclear | unclear | low | low | low | low | low | unclear |
| Tadokoro (2019)(46) | unclear | unclear | low | low | high | low | low | high |
| Tian (2019)(47) | low | low | low | low | low | low | low | low |
| Park (2020)(48) | low | unclear | unclear | unclear | unclear | low | low | unclear |
| Stavrinou (2020)(49) | low | unclear | low | low | unclear | low | low | unclear |
| Thapa (2020)(50) | low | unclear | unclear | unclear | low | low | low | unclear |

**Supplementary Table 5.** Effect size for all direct and indirect comparisons in MMSE change

| Comparator | Intervention | | | | | |
| --- | --- | --- | --- | --- | --- | --- |
|  | Estrogen | Anserine/carnosine supplementation | Triflusal | Fluoxetine | Rofecoxib |  |
| Estrogen |  | 1.00 (-2.89,4.89) | 0.59 (-3.16,4.33) | 2.63 (-1.47,6.73) | 0.10 (-3.60,3.80) |  |
| Anserine/carnosine supplementation | -1.00 (-4.89,2.89) |  | -0.41 (-4.07,3.25) | 1.63 (-2.39,5.65) | -0.90 (-4.51,2.71) |  |
| Triflusal | -0.59 (-4.33,3.16) | 0.41 (-3.25,4.07) |  | 2.04 (-1.84,5.92) | -0.49 (-3.95,2.97) |  |
| Fluoxetine | -2.63 (-6.73,1.47) | -1.63 (-5.65,2.39) | -2.04 (-5.92,1.84) |  | -2.53 (-6.37,1.31) |  |
| Rofecoxib | -0.10 (-3.80,3.60) | 0.90 (-2.71,4.51) | 0.49 (-2.97,3.95) | 2.53 (-1.31,6.37) |  |  |
| Cholinesterase inhibitor | -0.52 (-3.65,2.61) | 0.48 (-2.54,3.51) | 0.07 (-2.77,2.91) | 2.11 (-1.18,5.40) | -0.42 (-3.20,2.36) |  |
| Chinese herbal medicine | -1.50 (-4.56,1.56) | -0.50 (-3.45,2.45) | -0.91 (-3.67,1.85) | 1.13 (-2.09,4.35) | -1.40 (-4.10,1.30) |  |
| Antioxidant and unsaturated fatty acids | -2.90 (-7.08,1.28) | -1.90 (-6.01,2.21) | -2.31 (-6.28,1.66) | -0.27 (-4.58,4.04) | -2.80 (-6.73,1.13) |  |
| Unsaturated fatty acids | -1.85 (-5.32,1.62) | -0.85 (-4.23,2.53) | -1.26 (-4.48,1.96) | 0.78 (-2.84,4.40) | -1.75 (-4.91,1.42) |  |
| Antioxidant | -1.34 (-4.28,1.61) | -0.34 (-3.17,2.50) | -0.75 (-3.39,1.89) | 1.29 (-1.82,4.41) | -1.24 (-3.81,1.34) |  |
| Physical exercise and cognition-based intervention | -2.26 (-5.34,0.81) | -1.26 (-4.23,1.71) | -1.67 (-4.46,1.11) | 0.37 (-2.87,3.61) | -2.16 (-4.89,0.56) |  |
| Physical exercise | -2.32 (-5.21,0.58) | -1.32 (-4.10,1.47) | -1.73 (-4.31,0.86) | 0.31 (-2.76,3.39) | -2.22 (-4.73,0.30) |  |
| Cognition-based intervention and cholinesterase inhibitor | 0.36 (-3.31,4.02) | 1.36 (-2.22,4.93) | 0.94 (-2.48,4.37) | 2.99 (-0.82,6.79) | 0.46 (-2.92,3.83) |  |
| Cognition-based intervention | -1.20 (-4.11,1.71) | -0.20 (-3.00,2.59) | -0.61 (-3.21,1.98) | 1.43 (-1.65,4.51) | -1.10 (-3.63,1.43) |  |
| Psychosocial intervention | -1.68 (-4.96,1.61) | -0.68 (-3.87,2.51) | -1.09 (-4.10,1.93) | 0.95 (-2.49,4.39) | -1.58 (-4.54,1.38) |  |
| Health education | -1.38 (-4.37,1.60) | -0.38 (-3.26,2.50) | -0.79 (-3.48,1.89) | 1.25 (-1.91,4.40) | -1.28 (-3.90,1.34) |  |
| Control group | -0.40 (-3.21,2.41) | 0.60 (-2.09,3.29) | 0.19 (-2.29,2.67) | 2.23 (-0.76,5.22) | -0.30 (-2.71,2.11) |  |
|  |  |  |  |  |  |  |
|  | Cholinesterase inhibitor | Chinese herbal medicine | Antioxidant and unsaturated fatty acids | Unsaturated fatty acids | Antioxidant |  |
| Estrogen | 0.52 (-2.61,3.65) | 1.50 (-1.56,4.56) | 2.90 (-1.28,7.08) | 1.85 (-1.62,5.32) | 1.34 (-1.61,4.28) |  |
| Anserine/carnosine supplementation | -0.48 (-3.51,2.54) | 0.50 (-2.45,3.45) | 1.90 (-2.21,6.01) | 0.85 (-2.53,4.23) | 0.34 (-2.50,3.17) |  |
| Triflusal | -0.07 (-2.91,2.77) | 0.91 (-1.85,3.67) | 2.31 (-1.66,6.28) | 1.26 (-1.96,4.48) | 0.75 (-1.89,3.39) |  |
| Fluoxetine | -2.11 (-5.40,1.18) | -1.13 (-4.35,2.09) | 0.27 (-4.04,4.58) | -0.78 (-4.40,2.84) | -1.29 (-4.41,1.82) |  |
| Rofecoxib | 0.42 (-2.36,3.20) | 1.40 (-1.30,4.10) | 2.80 (-1.13,6.73) | 1.75 (-1.42,4.91) | 1.24 (-1.34,3.81) |  |
| Cholinesterase inhibitor |  | 0.98 (-0.84,2.81) | 2.38 (-1.02,5.78) | 1.33 (-1.14,3.80) | 0.82 (-0.73,2.37) |  |
| Chinese herbal medicine | -0.98 (-2.81,0.84) |  | 1.40 (-1.93,4.73) | 0.35 (-2.03,2.73) | -0.16 (-1.58,1.26) |  |
| Antioxidant and unsaturated fatty acids | -2.38 (-5.78,1.02) | -1.40 (-4.73,1.93) |  | -1.05 (-4.77,2.67) | -1.56 (-4.79,1.67) |  |
| Unsaturated fatty acids | -1.33 (-3.80,1.14) | -0.35 (-2.73,2.03) | 1.05 (-2.67,4.77) |  | -0.51 (-2.75,1.72) |  |
| Antioxidant | -0.82 (-2.37,0.73) | 0.16 (-1.26,1.58) | 1.56 (-1.67,4.79) | 0.51 (-1.72,2.75) |  |  |
| Physical exercise and cognition-based intervention | -1.75 (-3.61,0.12) | -0.76 (-2.51,0.98) | 0.64 (-2.71,3.99) | -0.42 (-2.82,1.99) | -0.93 (-2.43,0.58) |  |
| Physical exercise | -1.80 (-3.35,-0.25) | -0.81 (-2.22,0.59) | 0.58 (-2.60,3.77) | -0.47 (-2.64,1.70) | -0.98 (-2.06,0.10) |  |
| Cognition-based intervention and cholinesterase inhibitor | 0.87 (-1.50,3.25) | 1.86 (-0.79,4.51) | 3.26 (-0.64,7.16) | 2.20 (-0.92,5.33) | 1.69 (-0.80,4.19) |  |
| Cognition-based intervention | -0.69 (-2.26,0.89) | 0.30 (-1.13,1.73) | 1.70 (-1.50,4.89) | 0.65 (-1.54,2.83) | 0.13 (-1.02,1.29) |  |
| Psychosocial intervention | -1.16 (-3.36,1.04) | -0.18 (-2.27,1.92) | 1.22 (-2.32,4.77) | 0.17 (-2.50,2.84) | -0.34 (-2.25,1.57) |  |
| Health education | -0.87 (-2.58,0.85) | 0.12 (-1.46,1.70) | 1.52 (-1.75,4.79) | 0.47 (-1.82,2.75) | -0.05 (-1.37,1.28) |  |
| Control group | 0.12 (-1.27,1.50) | 1.10 (-0.11,2.32) | 2.50 (-0.60,5.60) | 1.45 (-0.60,3.49) | 0.94 (0.04,1.83) |  |
|  |  |  |  |  |  |  |
|  | Physical exercise and cognition-based intervention | Physical exercise | Cognition-based intervention and cholinesterase inhibitor | Cognition-based intervention | Psychosocial intervention |  |
| Estrogen | 2.26 (-0.81,5.34) | 2.32 (-0.58,5.21) | -0.36 (-4.02,3.31) | 1.20 (-1.71,4.11) | 1.68 (-1.61,4.96) |  |
| Anserine/carnosine supplementation | 1.26 (-1.71,4.23) | 1.32 (-1.47,4.10) | -1.36 (-4.93,2.22) | 0.20 (-2.59,3.00) | 0.68 (-2.51,3.87) |  |
| Triflusal | 1.67 (-1.11,4.46) | 1.73 (-0.86,4.31) | -0.94 (-4.37,2.48) | 0.61 (-1.98,3.21) | 1.09 (-1.93,4.10) |  |
| Fluoxetine | -0.37 (-3.61,2.87) | -0.31 (-3.39,2.76) | -2.99 (-6.79,0.82) | -1.43 (-4.51,1.65) | -0.95 (-4.39,2.49) |  |
| Rofecoxib | 2.16 (-0.56,4.89) | 2.22 (-0.30,4.73) | -0.46 (-3.83,2.92) | 1.10 (-1.43,3.63) | 1.58 (-1.38,4.54) |  |
| Cholinesterase inhibitor | 1.75 (-0.12,3.61) | 1.80 (0.25,3.35) | -0.87 (-3.25,1.50) | 0.69 (-0.89,2.26) | 1.16 (-1.04,3.36) |  |
| Chinese herbal medicine | 0.76 (-0.98,2.51) | 0.81 (-0.59,2.22) | -1.86 (-4.51,0.79) | -0.30 (-1.73,1.13) | 0.18 (-1.92,2.27) |  |
| Antioxidant and unsaturated fatty acids | -0.64 (-3.99,2.71) | -0.58 (-3.77,2.60) | -3.26 (-7.16,0.64) | -1.70 (-4.89,1.50) | -1.22 (-4.77,2.32) |  |
| Unsaturated fatty acids | 0.42 (-1.99,2.82) | 0.47 (-1.70,2.64) | -2.20 (-5.33,0.92) | -0.65 (-2.83,1.54) | -0.17 (-2.84,2.50) |  |
| Antioxidant | 0.93 (-0.58,2.43) | 0.98 (-0.10,2.06) | -1.69 (-4.19,0.80) | -0.13 (-1.29,1.02) | 0.34 (-1.57,2.25) |  |
| Physical exercise and cognition-based intervention |  | 0.05 (-1.11,1.21) | -2.62 (-5.29,0.05) | -1.06 (-2.34,0.22) | -0.59 (-2.42,1.25) |  |
| Physical exercise | -0.05 (-1.21,1.11) |  | -2.67 (-5.14,-0.21) | -1.11 (-2.01,-0.22) | -0.64 (-2.34,1.06) |  |
| Cognition-based intervention and cholinesterase inhibitor | 2.62 (-0.05,5.29) | 2.67 (0.21,5.14) |  | 1.56 (-0.92,4.04) | 2.03 (-0.88,4.95) |  |
| Cognition-based intervention | 1.06 (-0.22,2.34) | 1.11 (0.22,2.01) | -1.56 (-4.04,0.92) |  | 0.47 (-1.16,2.11) |  |
| Psychosocial intervention | 0.59 (-1.25,2.42) | 0.64 (-1.06,2.34) | -2.03 (-4.95,0.88) | -0.47 (-2.11,1.16) |  |  |
| Health education | 0.88 (-0.35,2.11) | 0.93 (-0.06,1.92) | -1.74 (-4.31,0.83) | -0.18 (-1.22,0.86) | 0.29 (-1.52,2.11) |  |
| Control group | 1.86 (0.60,3.12) | 1.92 (1.19,2.64) | -0.76 (-3.12,1.60) | 0.80 (0.04,1.57) | 1.28 (-0.43,2.99) |  |
|  |  |  |  |  |  |  |
|  | Health education | Control group |  |  |  |  |
| Estrogen | 1.38 (-1.60,4.37) | 0.40 (-2.41,3.21) |  |  |  |  |
| Anserine/carnosine supplementation | 0.38 (-2.50,3.26) | -0.60 (-3.29,2.09) |  |  |  |  |
| Triflusal | 0.79 (-1.89,3.48) | -0.19 (-2.67,2.29) |  |  |  |  |
| Fluoxetine | -1.25 (-4.40,1.91) | -2.23 (-5.22,0.76) |  |  |  |  |
| Rofecoxib | 1.28 (-1.34,3.90) | 0.30 (-2.11,2.71) |  |  |  |  |
| Cholinesterase inhibitor | 0.87 (-0.85,2.58) | -0.12 (-1.50,1.27) |  |  |  |  |
| Chinese herbal medicine | -0.12 (-1.70,1.46) | -1.10 (-2.32,0.11) |  |  |  |  |
| Antioxidant and unsaturated fatty acids | -1.52 (-4.79,1.75) | -2.50 (-5.60,0.60) |  |  |  |  |
| Unsaturated fatty acids | -0.47 (-2.75,1.82) | -1.45 (-3.49,0.60) |  |  |  |  |
| Antioxidant | 0.05 (-1.28,1.37) | -0.94 (-1.83,-0.04) |  |  |  |  |
| Physical exercise and cognition-based intervention | -0.88 (-2.11,0.35) | -1.86 (-3.12,-0.60) |  |  |  |  |
| Physical exercise | -0.93 (-1.92,0.06) | -1.92 (-2.64,-1.19) |  |  |  |  |
| Cognition-based intervention and cholinesterase inhibitor | 1.74 (-0.83,4.31) | 0.76 (-1.60,3.12) |  |  |  |  |
| Cognition-based intervention | 0.18 (-0.86,1.22) | -0.80 (-1.57,-0.04) |  |  |  |  |
| Psychosocial intervention | -0.29 (-2.11,1.52) | -1.28 (-2.99,0.43) |  |  |  |  |
| Health education |  | -0.98 (-2.01,0.04) |  |  |  |  |
| Control group | 0.98 (-0.04,2.01) |  |  |  |  |  |

**Supplementary Table 6.** Node-splitting model assessing incoherence between direct and indirect comparisons

|  | Comparisons | Effect size (Coef.±SE) | | | P | Tau |
| --- | --- | --- | --- | --- | --- | --- |
|  |  | Direct | Indirect | Difference |  |  |
| 1 | Control group vs Health education | -0.45±0.93 | 1.58±0.6 | -2.03±1.11 | 0.069 | 1.18 |
| 2 | Control group vs Cognition-based intervention | 0.66±0.45 | 1.25±0.79 | -0.59±0.91 | 0.513 | 1.23 |
| 3* | Control group vs Cognition-based intervention and cholinesterase inhibitor | -1.1±1.33 | 0.9±2.89 | -2±3.17 | 0.528 | 1.23 |
| 4 | Control group vs Physical exercise | 2.3±0.42 | 0.97±0.67 | 1.32±0.79 | 0.094 | 1.18 |
| 5* | Control group vs Antioxidant | 0.87±0.49 | 1.43±1.32 | -0.56±1.41 | 0.690 | 1.24 |
| 6* | Control group vs Chinese herbal medicine | 0.94±0.64 | 3.27±2.39 | -2.33±2.47 | 0.347 | 1.22 |
| 7* | Control group vs Cholinesterase inhibitor | 0±0.75 | 1.43±2.46 | -1.44±2.57 | 0.576 | 1.23 |
| 8 | Health education vs Cognition-based intervention | 0.41±0.77 | -0.71±0.73 | 1.11±1.06 | 0.293 | 1.22 |
| 9 | Health education vs Physical exercise | 0.45±0.79 | 1.26±0.66 | -0.81±1.03 | 0.433 | 1.23 |
| 10 | Health education vs Physical exercise and cognition-based intervention | 0.04±0.91 | 1.62±0.85 | -1.58±1.25 | 0.206 | 1.21 |
| 11* | Psychosocial intervention vs Cognition-based intervention | -0.13±0.91 | -2.28±2.08 | 2.15±2.27 | 0.343 | 1.22 |
| 12 | Psychosocial intervention vs Physical exercise | -0.1±1.25 | 1.34±1.22 | -1.44±1.74 | 0.409 | 1.23 |
| 13 | Psychosocial intervention vs Physical exercise and cognition-based intervention | 0.1±1.26 | 1.22±1.44 | -1.12±1.91 | 0.557 | 1.23 |
| 14 | Cognition-based intervention vs Physical exercise | 1.53±0.93 | 0.98±0.53 | 0.55±1.07 | 0.608 | 1.24 |
| 15 | Cognition-based intervention vs Physical exercise and cognition-based intervention | 0.5±1.26 | 1.27±0.78 | -0.77±1.48 | 0.602 | 1.23 |
| 16* | Cognition-based intervention and cholinesterase inhibitor vs Cholinesterase inhibitor | 0.5±1.36 | 2.5±2.85 | -2±3.17 | 0.528 | 1.23 |
| 17 | Physical exercise vs Physical exercise and cognition-based intervention | 0.7±0.71 | -1.49±0.98 | 2.19±1.2 | 0.069 | 1.18 |
| 18 | Physical exercise vs Antioxidant | -0.66±0.93 | -1.15±0.69 | 0.49±1.16 | 0.672 | 1.24 |
| 19 | Antioxidant vs Chinese herbal medicine | -0.07±1.26 | 0.29±0.9 | -0.36±1.55 | 0.818 | 1.24 |
| 20 | Antioxidant vs Cholinesterase inhibitor | -0.11±1.29 | -1.25±1.01 | 1.14±1.64 | 0.487 | 1.23 |

* All the evidence about these contrasts comes from the trials which directly compare them.


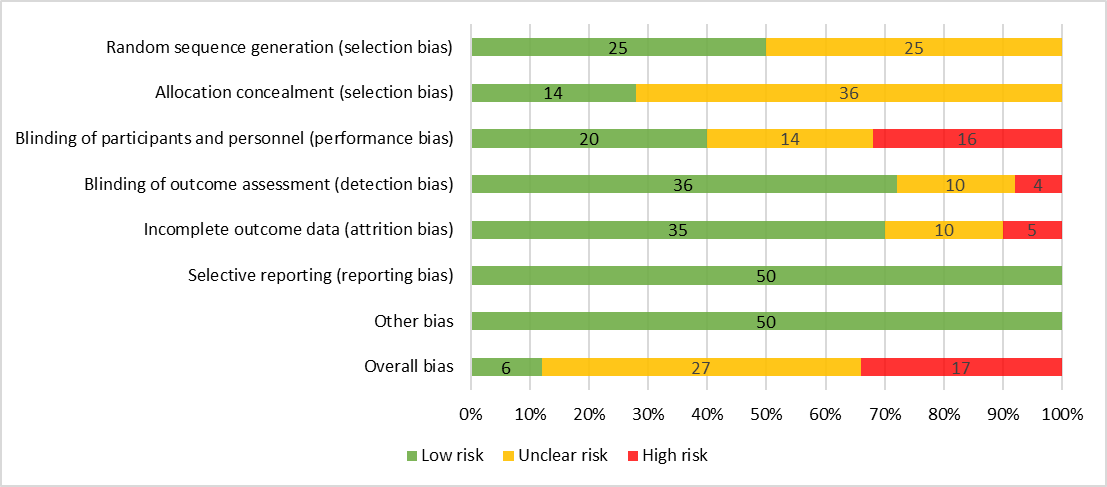


**Supplementary Figure 1.** Summary of risk of bias evaluated using the Cochrane Risk of Bias tool


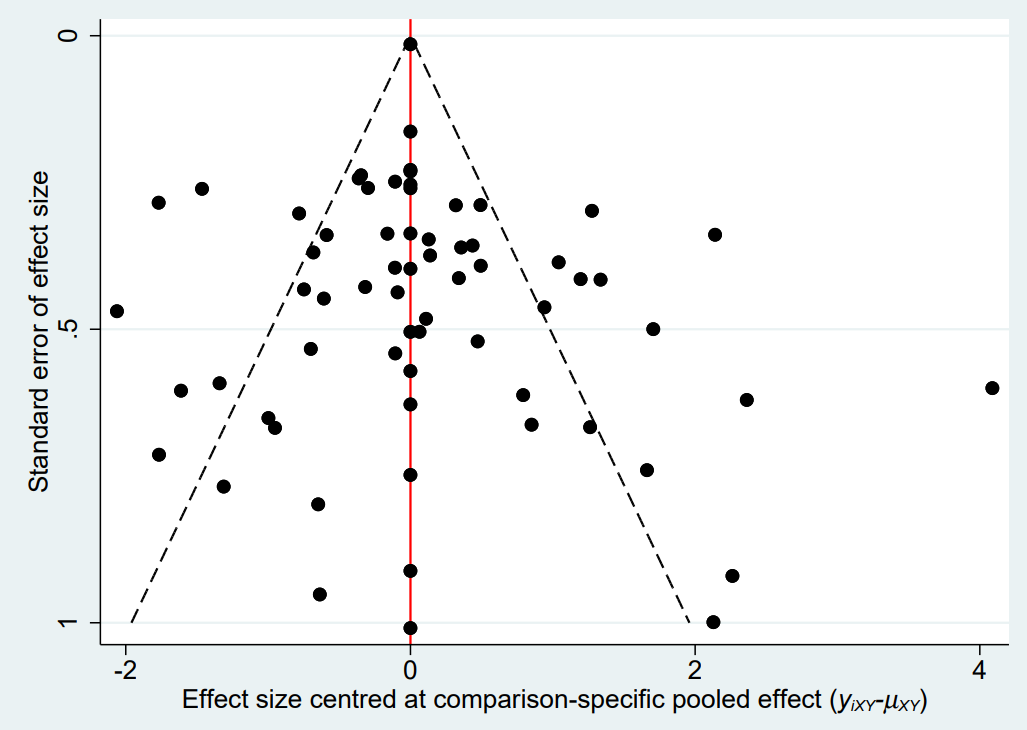


**Supplementary Figure 2.** Publication bias assessed via funnel plots

**References**

1. Petersen RC, Thomas RG, Grundman M, Bennett D, Doody R, Ferris S, et al. Vitamin E and donepezil for the treatment of mild cognitive impairment. *New England Journal of Medicine* (2005) 352(23):2379-468. PubMed PMID: 106552298. Corporate Author: Alzheimer's Disease Cooperative Study Group. Language: English. Entry Date: 20051209. Revision Date: 20161209. Publication Type: journal article.

2. Thal LJ, Ferris SH, Kirby L, Block GA, Lines CR, Yuen E, et al. A randomized, double-blind, study of rofecoxib in patients with mild cognitive impairment. *Neuropsychopharmacology* (2005) 30(6):1204‐15. doi: 10.1038/sj.npp.1300690. PubMed PMID: CN-00513662.

3. Mowla A, Mosavinasab M, Pani A. Does fluoxetine have any effect on the cognition of patients with mild cognitive impairment? A double-blind, placebo-controlled, clinical trial. *Journal of clinical psychopharmacology* (2007) 27(1):67‐70. doi: 10.1097/JCP.0b013e31802e0002. PubMed PMID: CN-00577728.

4. Rozzini L, Costardi D, Chilovi BV, Franzoni S, Trabucchi M, Padovani A. Efficacy of cognitive rehabilitation in patients with mild cognitive impairment treated with cholinesterase inhibitors. *International Journal of Geriatric Psychiatry* (2007) 22(4):356-60. PubMed PMID: 106171299. Language: English. Entry Date: 20071019. Revision Date: 20150711. Publication Type: Journal Article.

5. Gómez-Isla T, Blesa R, Boada M, Clarimón J, Del Ser T, Domenech G, et al. A randomized, double-blind, placebo controlled-trial of triflusal in mild cognitive impairment: the TRIMCI study. *Alzheimer Disease & Associated Disorders* (2008) 22(1):21-9. PubMed PMID: 105735455. Corporate Author: TRIMCI Study Group. Language: English. Entry Date: 20080606. Revision Date: 20150711. Publication Type: Journal Article.

6. Doody RS, Ferris SH, Salloway S, Sun Y, Goldman R, Watkins WE, et al. Donepezil treatment of patients with MCI: A 48-week randomized, placebo-controlled trial. *Neurology* (2009) 72(18):1555-61. doi: 10.1212/01.wnl.0000344650.95823.03. PubMed PMID: 105521788. Language: English. Entry Date: 20090717. Revision Date: 20150711. Publication Type: Journal Article.

7. Kwok TC, Bai X, Kao HS, Li JC, Ho FK. Cognitive effects of calligraphy therapy for older people: a randomized controlled trial in Hong Kong. *Clinical interventions in aging* (2011) 6:269‐73. doi: 10.2147/CIA.S25395. PubMed PMID: CN-00860581.

8. Sherwin BB, Chertkow H, Schipper H, Nasreddine Z. A randomized controlled trial of estrogen treatment in men with mild cognitive impairment. *Neurobiology of aging* (2011) 32(10):1808‐17. doi: 10.1016/j.neurobiolaging.2009.11.002. PubMed PMID: CN-00813597.

9. Rondanelli M, Opizzi A, Faliva M, Mozzoni M, Antoniello N, Cazzola R, et al. Effects of a diet integration with an oily emulsion of DHA-phospholipids containing melatonin and tryptophan in elderly patients suffering from mild cognitive impairment. *Nutritional Neuroscience* (2012) 15(2):46-54. doi: 10.1179/1476830511Y.0000000032.

10. Suzuki T, Shimada H, Makizako H, Doi T, Yoshida D, Tsutsumimoto K, et al. Effects of multicomponent exercise on cognitive function in older adults with amnestic mild cognitive impairment: a randomized controlled trial. *BMC Neurology* (2012) 12(1):128-. doi: 10.1186/1471-2377-12-128. PubMed PMID: 104307014. Language: English. Entry Date: 20130621. Revision Date: 20171010. Publication Type: journal article.

11. Varela S, Ayán C, Cancela JM, Martín V. Effects of two different intensities of aerobic exercise on elderly people with mild cognitive impairment: a randomized pilot study. *Clinical Rehabilitation* (2012) 26(5):442-50. doi: 10.1177/0269215511425835. PubMed PMID: 104553154. Language: English. Entry Date: 20120420. Revision Date: 20150711. Publication Type: Journal Article.

12. Lee L, Shahar S, Chin A-V, Yusoff N. Docosahexaenoic acid-concentrated fish oil supplementation in subjects with mild cognitive impairment (MCI): a 12-month randomised, double-blind, placebo-controlled trial. *Psychopharmacology* (2013) 225(3):605-12. doi: 10.1007/s00213-012-2848-0. PubMed PMID: 104336213. Language: English. Entry Date: 20130301. Revision Date: 20150711. Publication Type: Journal Article. Journal Subset: Biomedical.

13. Rojas GJ, Villar V, Iturry M, Harris P, Serrano CM, Herrera JA, et al. Efficacy of a cognitive intervention program in patients with mild cognitive impairment. *International Psychogeriatrics* (2013) 25(5):825-31. doi: 10.1017/S1041610213000045. PubMed PMID: 104256957. Language: English. Entry Date: 20130920. Revision Date: 20180807. Publication Type: journal article.

14. Suzuki T, Shimada H, Makizako H, Doi T, Yoshida D, Ito K, et al. A randomized controlled trial of multicomponent exercise in older adults with mild cognitive impairment. *Plos one* (2013) 8(4):e61483. doi: 10.1371/journal.pone.0061483. PubMed PMID: CN-00863231.

15. Yakoot M, Salem A, Helmy S. Effect of Memo®, a natural formula combination, on Mini-Mental State Examination scores in patients with mild cognitive impairment. *Clinical interventions in aging* (2013) 8:975‐81. doi: 10.2147/CIA.S44777. PubMed PMID: CN-00871926.

16. Zhang H, Zhao L, Yang S, Chen Z, Li Y, Peng X, et al. Clinical observation on effect of scalp electroacupuncture for mild cognitive impairment. *Journal of traditional chinese medicine = chung i tsa chih ying wen pan* (2013) 33(1):46‐50. doi: 10.1016/s0254-6272(13)60099-0. PubMed PMID: CN-00964262.

17. Johari SM, Shahar S, Ng TP, Rajikan R. A preliminary randomized controlled trial of multifaceted educational intervention for mild cognitive impairment among elderly Malays in Kuala Lumpur. *International journal of gerontology* (2014) 8(2):74‐80. doi: 10.1016/j.ijge.2013.07.002. PubMed PMID: CN-00995361.

18. Wei XH, Ji LL. Effect of handball training on cognitive ability in elderly with mild cognitive impairment. *Neuroscience letters* (2014) 566:98‐101. doi: 10.1016/j.neulet.2014.02.035. PubMed PMID: CN-00981093.

19. Zhang J, Wang Z, Xu S, Chen Y, Chen K, Liu L, et al. The effects of CCRC on cognition and brain activity in aMCI patients: a pilot placebo controlled BOLD fMRI study. *Current Alzheimer research* (2014) 11(5):484‐93. doi: 10.2174/1567205011666140505095939. PubMed PMID: CN-00994557.

20. Ciarmiello A, Gaeta MC, Benso F, Del Sette M. FDG-PET in the evaluation of brain metabolic changes induced by cognitive stimulation in aMCI subjects. *Current radiopharmaceuticals* (2015) 8(1):69‐75. PubMed PMID: CN-01084058.

21. Junying Z, Kai X, Dongfeng W, Rongjuan G, He L, Yongyan W, et al. The Effects of Bushen Capsule on Episodic Memory in Amnestic Mild Cognitive Impairment Patients: A Pilot Placebo Controlled fMRI Study. *Journal of Alzheimer's Disease* (2015) 46(3):665-76. doi: 10.3233/JAD-150004. PubMed PMID: 108817840. Language: English. Entry Date: 20160629. Revision Date: 20191029. Publication Type: journal article.

22. Lam LC, Chan WC, Leung T, Fung AW, Leung EM. Would older adults with mild cognitive impairment adhere to and benefit from a structured lifestyle activity intervention to enhance cognition?: a cluster randomized controlled trial. *PloS one* (2015) 10(3):e0118173. doi: 10.1371/journal.pone.0118173. PubMed PMID: CN-01171152.

23. Barban F, Annicchiarico R, Pantelopoulos S, Federici A, Perri R, Fadda L, et al. Protecting cognition from aging and Alzheimer's disease: a computerized cognitive training combined with reminiscence therapy. *International Journal of Geriatric Psychiatry* (2016) 31(4):340-8. doi: 10.1002/gps.4328. PubMed PMID: 113464723. Language: English. Entry Date: 20180728. Revision Date: 20191120. Publication Type: journal article.

24. Barekatain M, Alavirad M, Tavakoli M, Emsaki G, Maracy MR. Cognitive rehabilitation in patients with nonamnestic mild cognitive impairment. *Journal of research in medical sciences* (2016) 21(7). PubMed PMID: CN-01291788.

25. Giuli C, Papa R, Lattanzio F, Postacchini D. The Effects of Cognitive Training for Elderly: Results from My Mind Project. *Rejuvenation Research* (2016) 19(6):485-94. doi: 10.1089/rej.2015.1791. PubMed PMID: 120280727. Language: English. Entry Date: 20170411. Revision Date: 20180515. Publication Type: journal article.

26. Hagovská M, Olekszyová Z. Impact of the combination of cognitive and balance training on gait, fear and risk of falling and quality of life in seniors with mild cognitive impairment. *Geriatrics & Gerontology International* (2016) 16(9):1043-50. doi: 10.1111/ggi.12593. PubMed PMID: 117899111. Language: English. Entry Date: 20161007. Revision Date: 20170901. Publication Type: Article.

27. Jeong JH, Na HR, Choi SH, Kim J, Na DL, Seo SW, et al. Group- and Home-Based Cognitive Intervention for Patients with Mild Cognitive Impairment: A Randomized Controlled Trial. *Psychotherapy & Psychosomatics* (2016) 85(4):198-207. doi: 10.1159/000442261. PubMed PMID: 116320247. Language: English. Entry Date: 20170311. Revision Date: 20190711. Publication Type: journal article.

28. Zhang J, Liu Z, Zhang H, Yang C, Li H, Li X, et al. A Two-Year Treatment of Amnestic Mild Cognitive Impairment using a Compound Chinese Medicine: a Placebo Controlled Randomized Trial. *Scientific reports* (2016) 6:28982. doi: 10.1038/srep28982. PubMed PMID: CN-01600905.

29. Doi T, Verghese J, Makizako H, Tsutsumimoto K, Hotta R, Nakakubo S, et al. Effects of Cognitive Leisure Activity on Cognition in Mild Cognitive Impairment: Results of a Randomized Controlled Trial. *Journal of the American Medical Directors Association* (2017) 18(8):686-91. doi: 10.1016/j.jamda.2017.02.013. PubMed PMID: 124322242. Language: English. Entry Date: 20170807. Revision Date: 20191120. Publication Type: Article.

30. Hagovska M, Nagyova I. The transfer of skills from cognitive and physical training to activities of daily living: a randomised controlled study. *European Journal of Ageing* (2017) 14(2):133-42. doi: 10.1007/s10433-016-0395-y. PubMed PMID: 123085359. Language: English. Entry Date: 20170913. Revision Date: 20180601. Publication Type: Article.

31. Kohanpour MA, Peeri M, Azarbayjani MA. The Effects of Glycyrrhiza glabra L. extract use with aerobic training on inflammatory factors and cognitive state in elderly with mild cognitive impairment. *Journal of herbmed pharmacology* (2017) 6(4):178‐84. PubMed PMID: CN-01425538.

32. Kohanpour MA, Peeri M, Azarbayjani MA. The effects of aerobic exercise with lavender essence use on cognitive state and serum brain-derived neurotrophic factor levels in elderly with mild cognitive impairment. *Journal of herbmed pharmacology* (2017) 6(2):80‐4. PubMed PMID: CN-01366311.

33. Lazarou I, Parastatidis T, Tsolaki A, Gkioka M, Karakostas A, Douka S, et al. International Ballroom Dancing Against Neurodegeneration: A Randomized Controlled Trial in Greek Community-Dwelling Elders With Mild Cognitive impairment. *American Journal of Alzheimer's Disease & Other Dementias* (2017) 32(8):489-99. doi: 10.1177/1533317517725813. PubMed PMID: 126084327. Language: English. Entry Date: 20171114. Revision Date: 20171114. Publication Type: Article.

34. Poptsi E, Lazarou I, Markou N, Vassiloglou M, Nikolaidou E, Diamantidou A, et al. A comparative single-blind randomized controlled trial with language training in people with mild cognitive impairment. *American journal of alzheimer's disease and other dementias* (2018). PubMed PMID: CN-01690714.

35. Shimada H, Makizako H, Doi T, Park H, Tsutsumimoto K, Verghese J, et al. Effects of Combined Physical and Cognitive Exercises on Cognition and Mobility in Patients With Mild Cognitive Impairment: A Randomized Clinical Trial. *Journal of the American Medical Directors Association* (2018) 19(7):584-91. doi: 10.1016/j.jamda.2017.09.019. PubMed PMID: 130303895. Language: English. Entry Date: 20180628. Revision Date: 20190517. Publication Type: Article.

36. The effect of aerobic dance intervention on brain spontaneous activity in older adults with mild cognitive impairment: a resting-state functional MRI study. *Experimental and therapeutic medicine* (2019) 17(1):715‐22. doi: 10.3892/etm.2018.7006. PubMed PMID: CN-01925588.

37. Bademli K, Lok N, Canbaz M, Lok S. Effects of Physical Activity Program on cognitive function and sleep quality in elderly with mild cognitive impairment: A randomized controlled trial. *Perspectives in Psychiatric Care* (2019) 55(3):401-8. doi: 10.1111/ppc.12324. PubMed PMID: 137720415. Language: English. Entry Date: 20190730. Revision Date: 20190905. Publication Type: Article. Journal Subset: Core Nursing.

38. Bae S, Lee S, Lee S, Jung S, Makino K, Harada K, et al. The effect of a multicomponent intervention to promote community activity on cognitive function in older adults with mild cognitive impairment: A randomized controlled trial. *Complementary Therapies in Medicine* (2019) 42:164-9. doi: <https://doi.org/10.1016/j.ctim.2018.11.011>.

39. de Oliveira Silva F, Ferreira JV, Plácido J, Sant'Anna P, Araújo J, Marinho V, et al. Three months of multimodal training contributes to mobility and executive function in elderly individuals with mild cognitive impairment, but not in those with Alzheimer's disease: A randomized controlled trial. *Maturitas* (2019) 126:28-33. doi: 10.1016/j.maturitas.2019.04.217. PubMed PMID: 137127601. Language: English. Entry Date: In Process. Revision Date: 20191030. Publication Type: journal article. Journal Subset: Biomedical.

40. Klainin-Yobas P, Kowitlawakul Y, Lopez V, Tang CT, Hoek KE, Gan GL, et al. The effects of mindfulness and health education programs on the emotional state and cognitive function of elderly individuals with mild cognitive impairment: a randomized controlled trial. *Journal of clinical neuroscience* (2019) 68:211‐7. doi: 10.1016/j.jocn.2019.05.031. PubMed PMID: CN-01964484.

41. Langoni CdS, Resende TdL, Barcellos AB, Cecchele B, Knob MS, Silva TdN, et al. Effect of Exercise on Cognition, Conditioning, Muscle Endurance, and Balance in Older Adults With Mild Cognitive Impairment: A Randomized Controlled Trial. *Journal of Geriatric Physical Therapy* (2019) 42(2):E15-E22. doi: 10.1519/JPT.0000000000000191. PubMed PMID: 136136450. Language: English. Entry Date: 20190507. Revision Date: 20190516. Publication Type: Article. Journal Subset: Allied Health.

42. Masuoka N, Yoshimine C, Hori M, Tanaka M, Asada T, Abe K, et al. Effects of Anserine/Carnosine Supplementation on Mild Cognitive Impairment with APOE4. *Nutrients* (2019) 11(7):1626-. doi: 10.3390/nu11071626. PubMed PMID: 137681503. Language: English. Entry Date: 20190729. Revision Date: 20200116. Publication Type: Article. Journal Subset: Biomedical.

43. Park H, Park JH, Na HR, Hiroyuki S, Kim GM, Jung MK, et al. Combined intervention of physical activity, aerobic exercise, and cognitive exercise intervention to prevent cognitive decline for patients with mild cognitive impairment: a randomized controlled clinical study. *Journal of clinical medicine* (2019) 8 (7) (no pagination)(940). PubMed PMID: CN-01954531.

44. Park J, Kim SE, Kim EJ, Lee BI, Jeong JH, Na HR, et al. Effect of 12-week home-based cognitive training on cognitive function and brain metabolism in patients with amnestic mild cognitive impairment. *Clinical interventions in aging* (2019) 14:1167‐75. PubMed PMID: CN-01954587.

45. Park KC, Jin H, Zheng R, Kim S, Lee SE, Kim BH, et al. Cognition enhancing effect of panax ginsenin korean volunteers with mild cognitive impairment: a randomized, double-blind, placebo-controlled clinical trial. *Translational and clinical pharmacology* (2019) 27(3):92‐7. doi: 10.12793/tcp.2019.27.3.92. PubMed PMID: CN-01998329.

46. Tadokoro K, Morihara R, Ohta Y, Hishikawa N, Kawano S, Sasaki R, et al. Clinical Benefits of Antioxidative Supplement Twendee X for Mild Cognitive Impairment: a Multicenter, Randomized, Double-Blind, and Placebo-Controlled Prospective Interventionalfd Study. *Journal of Alzheimer's disease* (2019). doi: 10.3233/JAD-190644. PubMed PMID: CN-01989504.

47. Tian J, Shi J, Wei M, Ni J, Fang Z, Gao J, et al. Chinese herbal medicine Qinggongshoutao for the treatment of amnestic mild cognitive impairment: a 52-week randomized controlled trial. *Alzheimer's and dementia: translational research and clinical interventions* (2019) 5:441‐9. doi: 10.1016/j.trci.2019.03.001. PubMed PMID: CN-01990280.

48. Park JH, Liao Y, Kim DR, Song S, Lim JH, Park H, et al. Feasibility and tolerability of a culture-based virtual reality (VR) training program in patients with mild cognitive impairment: A randomized controlled pilot study. *International Journal of Environmental Research and Public Health* (2020) 17(9):3030. doi: <http://dx.doi.org/10.3390/ijerph17093030>.

49. Stavrinou PS, Andreou E, Aphamis G, Pantzaris M, Ioannou M, Patrikios IS, et al. The effects of a 6-month high dose omega-3 and omega-6 polyunsaturated fatty acids and antioxidant vitamins supplementation on cognitive function and functional capacity in older adults with mild cognitive impairment. *Nutrients* (2020) 12(2):325. doi: <http://dx.doi.org/10.3390/nu12020325>.

50. Thapa N, Park HJ, Yang JG, Son H, Jang M, Lee J, et al. The effect of a virtual reality-based intervention program on cognition in older adults with mild cognitive impairment: A randomized control trial. *Journal of Clinical Medicine* (2020) 9(5):1283. doi: <http://dx.doi.org/10.3390/jcm9051283>.
